# Sodium Content and Label Discrepancies in Processed and Ultra-Processed Foods in Bangladesh: A Public Health Concern

**DOI:** 10.3390/foods14213587

**Published:** 2025-10-22

**Authors:** Ummay Afroza, Ahmad Khairul Abrar, Abira Nowar, Sheikh Mohammad Mahbubus Sobhan, Abu Ahmed Shamim, Laura K Cobb, Nicole Ide, Sohel Reza Choudhury

**Affiliations:** 1 Department of Epidemiology and Research, National Heart Foundation Hospital and Research Institute, Dhaka 1216, Bangladesh; afrozatamanna.bd@gmail.com (U.A.); abrar.bd@gmail.com (A.K.A.); abiranowar0695@gmail.com (A.N.); smms_lincoln@yahoo.com (S.M.M.S.); ahmed.shamim@bracu.ac.bd (A.A.S.); 2 Resolve To Save Lives (RTSL), Alexandria, VA 22307, USA; lcobb@resolvetosavelives.org (L.K.C.); nide@resolvetosavelives.org (N.I.)

**Keywords:** processed food, ultra-processed food, sodium, salt, non-communicable disease, diet

## Abstract

Processed and ultra-processed foods (PF/UPFs) are becoming a significant public health concern because of their concerningly high nutrient content, including sodium, and rapidly increasing consumption, especially in low- and middle-income countries. This study aimed to analyze sodium levels in commonly consumed PF/UPFs in Bangladesh, compare them to the WHO’s sodium benchmarks, and assess the discrepancies between label-reported and laboratory-analyzed sodium content. A countrywide cross-sectional survey was conducted among adults, adolescents, and children to identify commonly consumed PFs/UPFs. Most common PF/UPFs were analyzed using Mohr’s titration method to estimate sodium content. Discrepancies between actual content and labels were analyzed, accepting a ±20% deviation, to determine gaps. Among the 974 participants surveyed, PF/UPF consumption in the past week was 97%, with higher consumption among metropolitan and urban residents. Amount of sodium, analyzed in 105 PF/UPF across 14 WHO categories and sub-categories, varied by categories, with soups, instant noodles, and chutneys having the highest. Compliance with the WHO’s sodium benchmarks was inconsistent across categories. The accuracy of label reporting was suboptimal, with under-reporting in 40% of products and a lack of sodium labelling in 9%. These findings unveiled high sodium intake from PFs/UPFs and inaccurate labelling as public health concerns in Bangladesh, highlighting the need for appropriate policies and strategies.

## 1. Introduction

Processed and ultra-processed foods (PF/UPFs) have been significant dietary energy sources in high-income countries (HICs) for decades [1]. Over the last decade, the availability of PFs/UPFs has increased globally, specifically in countries in Asia, and is primarily driven by food globalization [2,3]. According to the NOVA Food Classification System [4], processed foods (PFs) are defined as products made by adding salt, sugar, oil, or other substances to natural or minimally processed foods. Ultra-processed foods (UPFs), in contrast, are industrial formulations made entirely or mostly from substances extracted from foods (e.g., oils, fats, sugars, and starches) and from food constituents (e.g., hydrogenated fats and modified starch). While high-income countries continue to have higher proportions of PF/UPF consumption, low- and middle-income countries (LMICs) are experiencing the fastest growth in their consumption [5]. LMICs have undergone a phenomenon known as “Nutritional Transition,” characterized by a shift from traditional, homemade food consumption to energy-dense PFs/UPFs [2,3]. The transition occurs due to globalization, urbanization, economic development, unique marketing strategies, and technological advancement, which make these easy, appealing, and energy-dense foods more accessible and inexpensive [6]. Multiple studies indicate that these PFs/UPFs are low in dietary fibre and high in energy, sodium, sugar, and saturated fat, resulting in an increasing prevalence of obesity and other non-communicable diseases [7,8]. According to the Global Burden of Disease study, 2021, dietary risks were responsible for approximately 7.2 million deaths globally in 2021, making poor diet one of the leading risk factors for mortality worldwide [9,10].

Salt, chemically known as sodium chloride, is the primary source of dietary sodium. While sodium is physiologically essential, high sodium consumption is a major risk factor for the development of hypertension, which has been associated with stroke and coronary heart disease. A high-sodium diet is also associated with osteoporosis, renal disease, and gastric cancer [11,12,13,14]. Excess sodium consumption is one of the leading diet-related causes of death worldwide, causing approximately 1.86 million deaths per year [10].

Salt consumption in the Bangladeshi population ranges from 6.7 to 9 g/day [15], which is almost twice the World Health Organization’s (WHO) recommended maximum limit of 5 g of salt/day (equivalent to less than 2 g sodium/day) [16]. While homemade foods remain a primary source of dietary salt in Bangladesh [17], the increasing availability and consumption of PF/UPFs have introduced additional dietary salt exposure [18,19]. PF/UPFs often lack adequate nutritional labelling or provide inaccurate information on sodium content [20]. Research from Canada has identified significant differences between nutrition fact values and laboratory-measured values in packaged foods, with many exceeding 20% deviations [21]. This misinformation hinders consumers’ ability to make informed dietary choices regarding food and serves as an obstacle to reducing sodium intake [22].

Recognizing these challenges, the WHO has outlined evidence-based strategies for reducing sodium consumption through the “Best Buys” framework [23]. These include the reformulation of food products to contain less salt, implementation of an accurate and effective labelling system, restricting the marketing of unhealthy foods, the establishment of a healthy food-supportive environment in public or community settings, and promoting behaviour change communication and mass media campaigns [16,23]. In the Second National Plan of Action for Nutrition (2016–2025), the Bangladesh government outlined a multisectoral approach to reducing the consumption of processed food high in salt, sugar, and unhealthy fat through improved labelling practices and raising awareness and education, marketing restrictions, and social and behaviour change communication [24]. Shaheen et al. previously examined the consumption of processed packaged foods in Bangladesh and highlighted significant gaps in label conformity, reporting high sodium levels in these products. However, the study was limited in scope and included a smaller number of food items [20]. Building on these findings, this study aimed to determine the consumption of PF/UPFs, analyze the sodium content of commonly consumed PF/UPFs available in Bangladesh, evaluate their compliance with WHO sodium benchmarks, and investigate discrepancies between labelled and laboratory-measured sodium content.

## 2. Materials and Methods

This cross-sectional study included household and market surveys to identify commonly consumed processed and ultra-processed foods (PF/UPF) in Bangladesh. Selected products were analyzed in the laboratory to measure their sodium content and assess labelling accuracy.

### 2.1. Study Design and Setting

This cross-sectional study was conducted from December 2021 to April 2022 across all eight administrative divisions of Bangladesh. From each division, two districts were selected—one being the divisional headquarters and another one chosen randomly. In each district, three types of localities (metropolitan, urban, and rural) were surveyed, resulting in 24 survey clusters. Within each selected survey cluster, specific data collection locations were selected based on convenience.

In the household survey, all households in each area were listed, and random sampling was applied to select households and eligible participants (children 6–10 years, adolescents 11–18 years, and adults ≥ 19 years), ensuring equal representation by sex. People with food restrictions, cognitive impairment, and critical or terminal illnesses were excluded from the study.

For the market survey, 120 retail shops (5 per cluster) adjacent to the household survey locations were sampled, emphasizing larger retail shops with higher customer traffic and a wider variety of PFs/UPFs. Shops that were newer than 6 months and had fewer than 5 categories of PFs/UPFs were excluded.

### 2.2. Identification of Commonly Consumed Processed and Ultra-Processed Food

Household and market surveys were conducted to identify the commonly consumed PF/UPFs. Data collection was carried out by trained field research assistants and field supervisors using REDCap on Android tablet devices (Samsung Galaxy Tab A, model SM-T295; Samsung, Beijing, China). The household survey collected data on the frequency and types of PF/UPF consumed in the last seven days. Simultaneously, market surveys were conducted to assess the availability of labelled PF/UPF products through direct observation, interviews with shopkeepers regarding sales volume over the past 30 days, and one-hour customer purchasing observations during peak business hours.

Combining the results of household and market surveys, a list of PF/UPF products was prepared to identify commonly consumed PF/UPF. Commonly consumed food categories were identified (defined as categories that are available in more than 10% of shops, and an additional purposive category). From these selected categories, top-selling products were identified based on sales volumes of the last 30 days to ensure proportional representation across categories.

### 2.3. Sample Collection and Preparation

For the bromatological analysis, products with lower sodium content, such as milk-based chocolates, were excluded based on prior evidence of minimum sodium content, while candy was included. Products with higher sodium content (such as instant soups) were purposively included. Due to seasonal variation, we encountered challenges in obtaining certain products. For instance, ice cream was reported as frequently consumed in the household survey but was unavailable in shops during the winter sampling period. As a result, 105 products out of 120 under 16 categories were ultimately collected for analysis.

We collected samples of each PF/UPF from 16 out of 24 survey clusters. Trained field research assistants visited previously surveyed study sites and collected at least 12 samples of each product from the same food brand (e.g., 12 packets of instant noodles from brand “X”). These 12 samples were sourced from different clusters and at least 6 production batches to account for batch variability. In total, 1260 food samples were collected, representing 105 different PFs/UPFs products under 16 categories.

A brand-wise composite sample was prepared by pooling equal amounts from different production batches. Samples were immersed in liquid nitrogen to freeze thoroughly and create a crunchy mixture, and were then ground into powder. Approximately 100 g of each powdered sample was wrapped in aluminum foil, placed in an airtight bag, marked, and preserved at −20 °C until analysis.

### 2.4. Analysis of the Composite Samples

Salt content in food samples was determined using Mohr’s titrimetric method, a classical analytical technique for chloride estimation [25,26]. In this method, chloride ions in the sample were precipitated with a standard silver nitrate solution, using potassium chromate as an internal indicator. The endpoint was observed by the formation of a reddish-brown coloration due to the formation of silver chromate. The volume of silver nitrate consumed was recorded and used to calculate the salt concentration in the sample [27].

The percent NaCl was calculated using the following formula:

$$\%NaCl=\frac{Titre\;Value*Normality\;of\;the\;AgNO3*58.4*100}{Weight\;of\;the\;Sample*1000}$$

The calculated salt content was further converted to sodium using the conversion formula. Mohr’s titration method measured chloride instead of sodium to avoid the influence of sodium-containing food additives (e.g., sodium bicarbonate and sodium benzoate) [28]. This method is a widely accepted and cost-effective method [29], endorsed by food quality monitoring bodies such as the Food Safety and Standard Authority of India (FSSAI) [27], and the Codex Alimentarius recommended titration method to estimate salt content [30].

Although the method cannot account for non-chloride sodium, results are comparable to direct sodium determination techniques, like atomic absorption spectrophotometry (AAS) [31,32], making it suitable for reliable salt estimation in this study.

### 2.5. Processed and Ultra-Processed Food Categories

We collected data according to local conventional categories of PFs/UPFs. Later, they were classified based on the World Health Organization’s (WHO) recommended categories and sub-categories, following the WHO South-East Asia Region (SEARO) Sodium Benchmarks for Packaged Foods [25]. After recategorization in line with the WHO recommendations, the original 37 local categories were consolidated into 9 main categories and 14 sub-categories.

### 2.6. Statistical Analysis

We only conducted the descriptive analyses for this study. Results were presented as mean, median, and proportion, as appropriate. Means were presented with a 95% confidence interval. Data were analyzed using SPSS v.26.

#### 2.6.1. Sodium Content and Compliance with the WHO Benchmark

We reported category-wise mean and median amounts of laboratory-analyzed sodium values following the WHO categories and subcategories and compared the median values with the WHO SEARO Sodium Benchmark [33]. Compliance was assessed by determining the proportion of products with sodium levels below the WHO sodium target for the respective category.

In case of the categories having an interim target in the SEARO benchmark, we assessed compliance with the interim target. The WHO global sodium benchmarks [34] were used where SEARO targets were unavailable. The WHO did not set any targets for some categories and subcategories; we mentioned those accordingly.

#### 2.6.2. Calculation of Dietary Contribution of Sodium per Serving

Dietary contribution of sodium per serving PF/UPF was calculated according to the serving size reported on the package label. If the serving size was not reported, we used the median serving size of the respective food category for analysis.

Percent contribution from a single serving of PF/UPF to the recommended daily amount (RDA) of sodium was calculated based on the WHO-recommended daily sodium limit of 2000 mg. We used the following formula to calculate the percent contribution:

$$Percent\;\left(\%\right)\;sodium\;contribution=\frac{Amount\;of\;sodium\;per\;serving\;(\mathrm{mg})}{WHO\;recommended\;value\;(2000\;\mathrm{mg})}\times 100$$

#### 2.6.3. Comparison of Laboratory-Analyzed and Label-Declared Sodium Contents

We compared the laboratory-analyzed sodium amount with the corresponding sodium value reported on the package label using a ±20% threshold to determine under- or over-reporting. As the package labels’ reporting was diverse in terms of salt or sodium, reporting unit of per 100 g of food, per serving, or per package, we converted all label-reported values to sodium per 100 g of food, following the appropriate methods and standard conversion factor of 1 g sodium (Na) = 2.54 g salt (NaCl) was applied [34].

## 3. Results

### 3.1. Sample Characteristics

Among the 974 individuals interviewed in the household survey, one-fourth were children (6–10 years), one-fourth were adolescents (11–18 years), and half were adults (19 years and above), equally from metropolitan (34%), urban (33%), and rural (33%) areas. Half of the participants (50%) were female. The mean age of children was 8.1 years, the age of adolescents was 14.6 years, and the age of adults was 40.1 years. Almost half (49%) of the participants were students, one-fourth were housewives, and the rest were from other professions.

### 3.2. Processed and Ultra-Processed Foods Consumption

**Figure 1:**
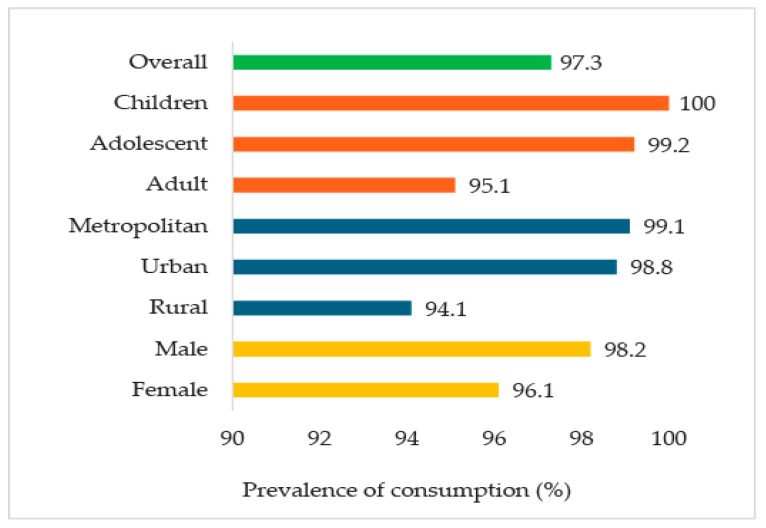
Prevalence of PF/UPF consumption.

The survey revealed a remarkably high prevalence of processed and ultra-processed food (PF/UPFs) consumption, with 97% of respondents reporting an intake of at least one PF/UPF product within the past week. It was found that every child and nearly all adolescents consumed PF/UPF at least once in the past week, with consumption rates substantially higher than those observed among adults (95%). Furthermore, a significant disparity in consumption patterns was observed between geographical regions, with metropolitan and urban residents displaying higher rates of PF/UPF consumption compared to their rural counterparts (Figure 1).

**Figure 2:**
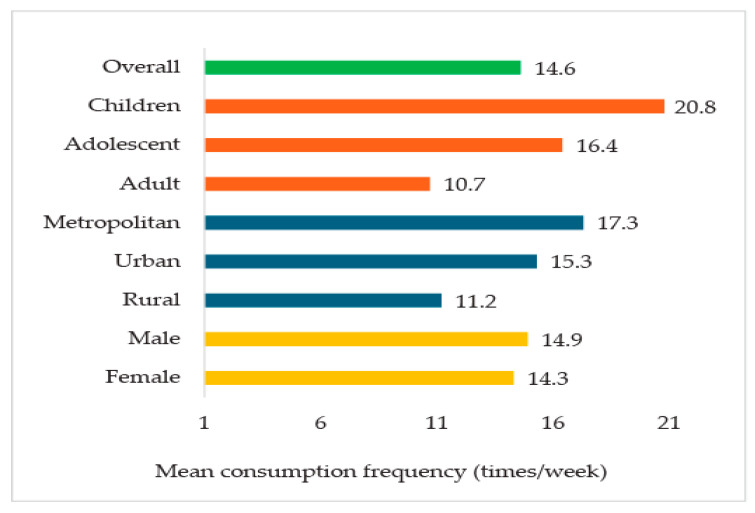
Consumption frequency of PF/UPF in a week.

Specifically, children consumed PF/UPF approximately 21 times per week, adolescents 16 times per week, and adults 11 times per week. Moreover, consumption frequency was notably higher in metropolitan areas, with an average of 17 times per week, compared to 11 times per week in rural areas (Figure 2).

The household survey identified 37 food categories of PF/UPFs. Simultaneously, 120 of the retail shops surveyed identified 825 labelled products across the different PF/UPF categories. Combining the results of the household and market survey, a list of PF/UPF products was prepared to identify commonly consumed PF/UPF. Of the 37 food categories identified, 16 were selected as commonly consumed: the top 15 categories were available in more than 10% of shops, and instant soup (purposively added due to its high sodium content). From these categories, 120 top-selling PF/UPF products were identified as commonly consumed. The three most frequently consumed PF/UPF categories were sweet biscuits (60.5%), chanachur (made by mixing various ingredients, such as wheat or pulse flour chips, fried peas, peanuts, rice, and spices) (53.07%), and instant noodles (52.25%).

### 3.3. Mean Sodium Content and Proportion of Products Meeting the Sodium Targets

Analysis of fourteen of the WHO’s sub-categories under eight of the WHO’s categories, which correspond to sixteen local food categories, revealed varying sodium levels per 100 g of food products. Soups exhibited the highest sodium content at 3149.6 mg/100 g, followed by instant noodles (1515.8 mg/100 g) and pickles (1378.6 mg/100 g). High sodium levels were also found (838.6 mg/100 g) in highly processed breakfast cereals (e.g., jhalmuri is a type of puffed rice mixed with spices). Chocolate (807.1 mg) and extruded snacks (chanachur) at 852.0 mg/100 g also have a high amount of salt. On the other hand, beverages (soft drinks and fruit-flavoured drinks) demonstrated the lowest sodium content at 9.8 mg/100 g (Table 1).

Comparing these values to the WHO Southeast Asia Region (SEARO) sodium benchmarks (which indicate maximum limits), the proportion of food items that meet the targets varies considerably between various food categories. Generally, savoury snacks such as extruded snacks and potato, vegetable, and grain chips show weak adherence; only 18.2% of potato, vegetable, and grain chips comply with these targets, whereas there are no extruded snacks. Contrarily, some sub-categories, such as crackers and savoury biscuits, have higher levels of compliance; 69.2% of savoury biscuits (salted biscuits and melba toast or rusks) meet the targets set by the WHO’s SEARO. Instant noodles have high levels of adherence (80%), while soups do not conform to them. Compliance is also varied in condiments and chutneys, where 50% of chutneys meet the benchmarks, but all condiments comply. Just over 60% of sweet baked goods, such as cakes and sponge, adhere to the benchmark, compared to only about a quarter (27.3%) of cookies/sweet biscuits. Leavened bread is entirely consistent with the sodium limit.

Overall, Table 1 indicates that 63% of the 105 analyzed products exceed the sodium limits according to the WHO SEARO Benchmarks or the WHO global benchmark. Although 37% of products meet the benchmark, they are still very high in sodium.

### 3.4. Per-Serving Sodium Content and Dietary Contribution

**Figure 3:**
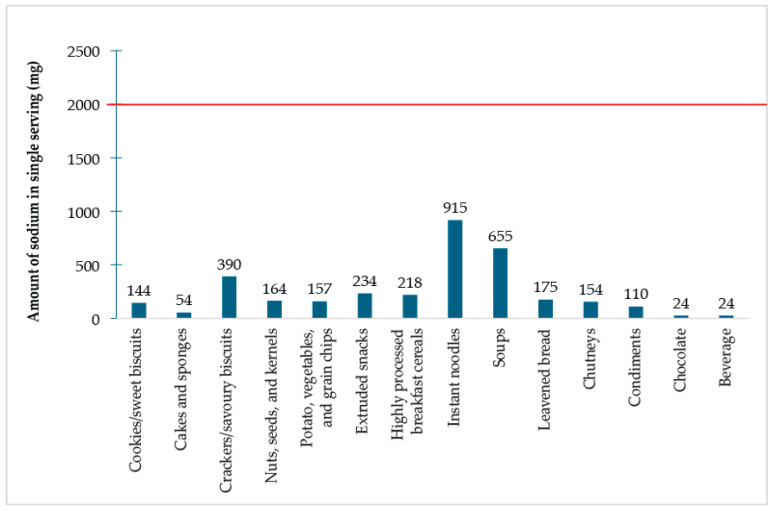
Per-serving sodium content of PF/UPF. The horizontal line indicates the daily maximum allowable intake of 2000 mg of sodium as recommended by the WHO.

The per-serving sodium content of PF/UPF was analyzed against the maximum daily allowable limit of 5 g per serving, as recommended by the WHO (Figure 3). None of the PF/UPF exceeds the threshold. Among all the foods, a single serving of instant noodles accounted for the highest amount of sodium (915 mg), followed by soups (655 mg) and crackers/savoury biscuits (390 mg). Conversely, chocolate and beverages had a minimal contribution, each adding only 24 mg of sodium per serving.

**Figure 4:**
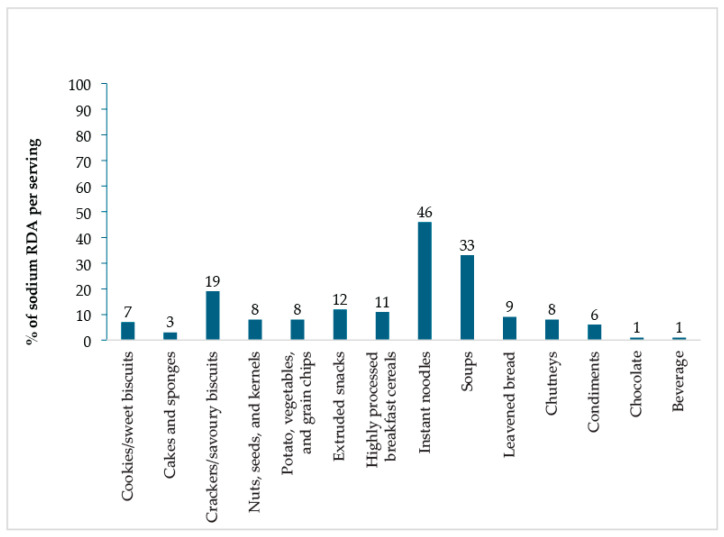
Percentage of daily sodium RDA contributed by a single serving of PF/UPF.

In terms of dietary recommendations for a 2000 Kcal diet, which allows a maximum of 2000 mg of sodium per day, some PF/UPF categories contributed significantly to the recommended daily allowance (RDA) (Figure 4). For example, a single serving of instant noodles and soups can meet 46% and 33% of the daily sodium limit, respectively. Moderately contributing foods are crackers/savoury biscuits (19.5%), extruded snacks (12%), and highly processed breakfast cereals (11%). In contrast, beverages and chocolate contribute minimally to the daily sodium intake, each providing about 1% (Figure 4).

### 3.5. Comparison of Laboratory-Measured and Label-Declared Sodium Content

A comparison of the laboratory-measured sodium content with the values reported on packaging labels is presented in Table 2. The results reveal discrepancies between declared and actceptable sodium levels, underscoring potential inaccuracies in product labelling. Overall, 40% of PF/UPF under-reported their sodium content, while 24% over-reported. Additionally, 9% of products lacked any sodium-related labelling.

Under-reporting was most frequently observed in nuts, seeds, and kernels (80%), potato, vegetables, and grain chips (64%), and instant noodles (60%). In contrast, condiments showed a tendency to over-report sodium content. Notably, all chocolate products and half of the leavened bread products lacked sodium labelling, which prevented assessment of their actual sodium content.

There was notable variability in labelling accuracy across food categories, with some categories demonstrating consistent trends toward either under-reporting or over-reporting. For example, cakes and sponges showed a higher tendency toward over-reporting (50%), while crackers and savoury biscuits exhibited more under-reporting (53.8%).

## 4. Discussion

The study was undertaken to determine the consumption patterns of processed and ultra-processed foods (PF/UPFs) in Bangladesh. It determined the sodium content of more than a hundred commonly consumed PF/UPFs, compared it with the WHO SEARO Sodium Benchmark, estimated their contribution to daily sodium intake, and identified discrepancies in label reporting for sodium content. This study provided a detailed analysis of sodium levels in commonly consumed PF/UPFs in Bangladesh. This study generated significant evidence for policymakers on the existing compliance gap in label reporting for sodium. It also added new insights into the potential health risks of high sodium consumption in Bangladeshi populations, especially among children and adolescents, by measuring the contribution of PF/UPFs to overall sodium intake.

The study’s findings revealed an alarmingly high prevalence of PF/UPF consumption in Bangladesh, with 97% of respondents reporting intake in the past week. This trend was particularly pronounced among children (100%) and adolescents (99.2%), who consumed PFs/UPFs approximately 21 and 16 times per week, respectively. These findings are consistent with our observed high consumption prevalence of PF/UPFs, particularly among children and adolescents. These findings also align with global trends observed in low- and middle-income countries (LMICs), where urbanization, globalization, and economic development have driven a nutritional transition toward energy-dense processed foods [35]. Previous studies in Bangladesh, such as one reporting that 83% of adolescents consume at least one ultra-processed or deep-fried item daily [18], and another reported frequent consumption of ready-to-eat processed foods [19]. A similar trend was observed in the neighbouring country, India, where 94.3% of children consumed salted packaged food [36], further supporting these findings. The rapid growth of Bangladesh’s processed food market, valued at USD 19.7 billion in 2018 and growing by 15% annually, reflects these consumption patterns [37] and is fueled by factors such as population growth, urbanization, widespread marketing, higher income levels, and increased food diversity [38,39]. As Bangladesh advances toward a high–middle-income country [40], the consumption of PF/UPFs is expected to rise further, underscoring the need for urgent public health interventions to address the associated health risks, including obesity and chronic diseases linked to PF/UPF consumption.

The sodium analysis revealed significant variations across food categories. Soups (3149.6 mg/100 g), instant noodles (1515.8 mg/100 g), and chutneys (1378.6 mg/100 g) were identified as the highest contributors to dietary sodium intake. These results are consistent with regional data from India, where similar products also exhibit high sodium content [41]. Compliance with the WHO Southeast Asia Regional Sodium Benchmarks varied widely among food categories. While 80% of instant noodles met the benchmarks, nuts, seeds, and kernels, extruded snacks, highly processed breakfast cereals, and soups showed no compliance. Similar findings were reported from Malaysia, where 90% of instant noodles exceeded the WHO’s recommended daily sodium intake [42], and in India, where many packaged foods surpass recommended sodium levels [43]. These findings highlight regulatory gaps and the need for stricter monitoring. As emphasized by Trieu et al., legislative action is crucial for reducing the level of sodium intake in the population [44].

Importantly, a single serving of some PF/UPFs provided a substantial portion of the WHO-recommended maximum allowable sodium limit. Among the analyzed foods, one serving of instant noodles covered half of the daily allowable sodium limit, whereas instant soup covered one-third. While this observation is consistent with findings in other LMICs [45], it highlights an urgent need for targeted interventions in Bangladesh to limit excessive sodium consumption.

A key finding of this study was the significant discrepancy between laboratory-measured sodium content and package labels’ declared values. Among the analyzed products, 40% under-reported sodium content beyond the acceptable deviation threshold (>20%). On the other hand, 24% of the products over-reported sodium levels, and 9% did not list sodium content at all. Despite regulations, such as Bangladesh’s Packaged Food Labelling Regulation 2017, mandating accurate nutrient declaration, compliance remains poor [46]. This ongoing non-compliance has also been documented in earlier research from Bangladesh [20], and similar challenges have been documented in the South Asian region, including India, where nutrient labels often fail to accurately report sodium levels [43]. These discrepancies highlight systemic enforcement failures that undermine consumer trust and hinder efforts to reduce dietary sodium intake.

Setting mandatory maximum sodium levels for packaged foods is an effective intervention to reduce population sodium intake. These targets set enforceable limits on sodium content, requiring manufacturers to reformulate products when they fall above the limits. The WHO’s global sodium benchmarks, adapted regionally for South-East Asia, provide a practical framework for developing such policies [47,48]. Evidence consistently shows that mandatory sodium targets outperform voluntary approaches [44,47,49]. A recent systematic review by Gressier et al. further confirms that mandatory reformulation policies achieve greater and more consistent reductions in harmful nutrients, such as sodium, compared to voluntary measures [50]. Moreover, modelling in Australia estimated that full compliance with the WHO’s benchmarks could reduce sodium intake by 12% and prevent nearly 1770 deaths annually [51]. Countries like South Africa, Argentina, and Colombia have developed policies mandating comprehensive sodium reduction targets. Evaluations in Argentina have shown strong compliance [52], and in South Africa, sodium consumption has decreased by 1.15 g/day since the policy was implemented [53].

Front-of-package warning labels (FOPWL) complement sodium targets by raising consumer awareness of foods that remain high in sodium and other nutrients of concern, and further motivate industry reformulation. For example, Chile’s FOPWL policy led to a 13.8% reduction in high-sodium food purchases [54]. In addition to reducing purchases, studies from Chile, Mexico, and Peru demonstrate that the industry reformulated food products to reduce sodium content to avoid the FOPWL [55,56,57]. Adopting such measures in Bangladesh could enhance transparency and drive healthier dietary choices.

Mandatory nutrition labelling is important in enhancing diets by ensuring consumers have access to essential information for informed choices, thereby promoting healthier eating habits [58,59,60]. While accurate back-of-pack (BOP) declarations are important, particularly as a pre-requisite for policy development, monitoring, and evaluation, evidence from Bangladesh and studies around the world suggests that FOPL, particularly warning labels, are perceived as easier to understand across diverse populations and more likely to influence consumer behaviour than other formats [61]. To enhance public health outcomes, Bangladesh should adopt a rigorous, government-endorsed FOPL system with strong enforcement measures, addressing existing labelling inconsistencies and fostering greater consumer awareness [20,54,61]. Such evidence-based interventions are particularly vital in LMICs to combat the growing burden of non-communicable diseases [45].

This study had several limitations. The primary limitation is that selected categories of food (16 in number) and food products (105 in number) might not be enough to represent the comprehensive diversity of PF/UPFs consumed in Bangladesh. Some frequently consumed foods remained out of the top list because of seasonal variation, and we had limited resources to cover more. In addition, the relatively small sample size, especially within some sub-categories, resulted in wide confidence intervals and limited the statistical power of the findings. Another limitation is that this study uses Mohr’s titration method for estimating sodium content. While this method is cost-effective and widely accepted, it only measures sodium indirectly through chloride levels, potentially underestimating total sodium content from other sodium-containing compounds, such as preservatives and leavening agents. Moreover, the statistical analysis was primarily descriptive, which, while suitable for the study’s exploratory nature, restricts inferential interpretation. Future studies should consider including a larger and more diverse range of food categories and consider employing more direct methods of sodium estimation.

This study has several notable strengths. It followed a robust method for identification of the commonly consumed PF/UPFs, surveying randomly selected representative samples covering metropolitan, urban, and rural areas of all eight administrative divisions of Bangladesh. The wide geographic coverage and random representative sampling enhanced the generalizability of the study findings related to identifying commonly consumed PF/UPFs in the national context. Another notable strength is the application of novel methodology for identifying the top-selling PF/UPFs in the absence of direct sales data. We used a rapid and simple method, combining the structured interviews of shopkeepers and direct customer observations to ensure the reflection of actual consumer behaviours. Another strength of this study is that it is the first study to evident label discrepancies for sodium content reporting by different PF/UPF categories. While the sodium analysis was conducted on a limited number of samples, it provides valuable exploratory evidence that can inform future, larger-scale investigations.

## 5. Conclusions

High sodium levels in PF/UPFs represent a significant public health challenge in Bangladesh, necessitating immediate action to reduce the risk of diet-related diseases and improve overall health. These findings would facilitate policymakers in the adoption of evidence-based measures to ensure a healthy dietary environment for the reduction in non-communicable disease burden, including mandatory sodium targets, labelling compliance, and stronger enforcement. A multi-pronged approach, including maximum sodium limits, mandatory FOPL, public education campaigns, and robust monitoring mechanisms, is essential to create a healthier food environment. Drawing lessons from global best practices, such as Chile’s FOPL regulation and Finland’s sodium reduction policies, can guide Bangladesh in developing effective strategies. Further research should explore the effectiveness of these interventions and monitor changes in consumption patterns over time to ensure sustainable health outcomes.

## Figures and Tables

**Table 1:** Comparison of sodium content against the WHO South-East Asia Regional (SEARO) Benchmark (*n* = 105).

Main Food Category and Subcategories	Local Name (*n*)	Median Sodium, mg/100 g	Mean Sodium (95% CI), mg/100 g	Minimum-Maximum Sodium, mg/100 g	SEARO/Global Sodium Benchmark, mg/100 g	Products Meeting Benchmark, *n* (%)
Cakes, sweet biscuits and pastries; other sweet bakery wares; and dry-mixes for making such						
Cookies/sweet biscuits (2a)	*Sweet biscuits* (33)	216.5	235.6 (205.9, 265.3)	98.4−555.1	200 **	9 (27.3)
Cakes and sponges (2b)	*Cake* (8)	196.9	184.6 (151.8, 217.3)	122.1−240.2	205 **	5 (62.5)
Savoury snacks						
Crackers/savoury biscuits (3a)	*Salted Biscuits* (8)	468.5	543.0 (411.1, 674.9)	322.8−1031.5	600	9 (69.2)
	*Toast Biscuits/ Rusk* (5)					
Nuts, seeds, and kernels (3b)	*Fried peas, pulses* (5)	633.9	624.4 (492.6, 756.2)	492.1−752.0	430 *	0
Potato, vegetable, and grain chips (3c)	*Chips* (11)	716.5	724.8 (571.2, 878.3)	358.3−1094.5	500	2 (18.2)
Extruded snacks (3d)	*Chanachur* (5)	811.0	852.0 (738.1, 965.9)	771.7−976.4	520	0
Breakfast cereals						
Highly processed breakfast cereals (6b)	*Jhalmuri* (2)	838.6	838.6 (688.5, 988.7)	826.8−850.4	280 **	0
Ready-made and convenience foods and composite dishes						
Instant noodles with sauce or seasoning (dry-mix, concentrated) (9biii)	*Instant Noodles* (5)	1476.4	1515.8 (1165.7, 1865.8)	1263.8−1968.5	1730 *	4 (80)
Soups (dry soup only) (concentrated) (9gii)	*Instant Soup* (3)	3740.2	3149.6 (506.4, 5792.8)	1921.3−3787.4	1200 **	0
Bread, and ordinary bakery wares						
Leavened bread (11b)	*Bread* (4)	318.9	314.0 (244.1, 383.8)	255.9−362.2	445 *	4 (100)
Sauces, dips and dressings						
Chutneys (involving brining or fermentation) (18ci)	*Pickles and Chutneys* (6)	944.9	1378.6 (−40.5, 2797.7)	283.5−4039.4	1000 *	3 (50)
Condiments (18e)	*Sauce* (3)	551.2	589.2 (165.6, 1012.9)	440.9−775.6	1005 *	3 (100)
Chocolate and sugar confectionary, energy bars, and sweet toppings and desserts						
Chocolate	*Candy* (2)	807.1	807.1 (−1444.0, 3058.2)	629.9−984.3	# Target not included in benchmarks	-
Beverage						
	*Soft drinks* (3)	7.87	9.8 (2.5, 17.2)	1.97−15.8	# Target not included in benchmarks	-
	*Fruit-flavored drinks* (2)					-

Note: Numbers in parentheses (e.g., 3a, 6b) refer to the serial numbers of the categories in the SEARO/Global Sodium Benchmark. * Indicates that the interim target of the SEARO benchmark was used. ** Indicates that the Global Sodium Benchmark category, subcategories, and targets were used. # Indicates that no target for the specific category or subcategory was included in the SEARO/Global Sodium Benchmark.

**Table 2:** Comparison of reported sodium content on package labels with measured sodium content.

Food Category	Under Reporting	Over Reporting	Acceptable Reporting	Reporting Absent
Cookies/sweet biscuits (33)	12 (36.4%)	9 (27.3%)	12 (36.4%)	0
Cakes and sponges (8)	2 (25.0%)	4 (50.0%)	2 (25.0%)	0
Crackers/savoury biscuits (13)	7 (53.8%)	2 (15.4%)	2 (15.4%)	2 (15.4%)
Nuts, seeds, and kernels (5)	4 (80.0%)	1 (20.0%)	0	0
Potato, vegetables, and grain chips (11)	7 (63.6%)	1 (9.1%)	2 (18.2%)	1 (9.1%)
Extruded snacks (5)	0	1 (20.0%)	4 (80.0%)	0
Highly processed breakfast cereals (2)	1 (50.0%)	0	1 (50.0%)	0
Instant noodles (5)	3 (60.0%)	0	1 (20.0%)	1 (20.0%)
Soups (3)	0	2 (66.7%)	1 (33.3%)	0
Leavened bread (4)	1 (25.0%)	0	1 (25.0%)	2 (50.0%)
Chutneys (6)	3 (50.0%)	1 (16.7%)	1 (16.7%)	1 (16.7%)
Condiments (3)	0	3 (100%)	0	0
Chocolate (2)	0	0	0	2 (100%)
Beverage (5)	2 (40.0%)	1 (20.0%)	2 (40.0%)	0
Total (105)	42 (40.0%)	25 (23.8%)	29 (27.6%)	9 (8.6%)

Note: A 20% deviation from the reported value was considered as acceptable reporting.

## Data Availability

The original contributions presented in the study are included in the article; further inquiries can be directed to the corresponding author.

## Abbreviations

The following abbreviations are used in this manuscript:

PF/UPF	Processed and ultra-processed foods
LMICs	Low and middle-income countries
HICs	High-income countries
CVD	Cardiovascular disease
WHO	World Health Organization
SEARO	South-East Asia Region
